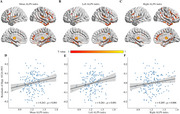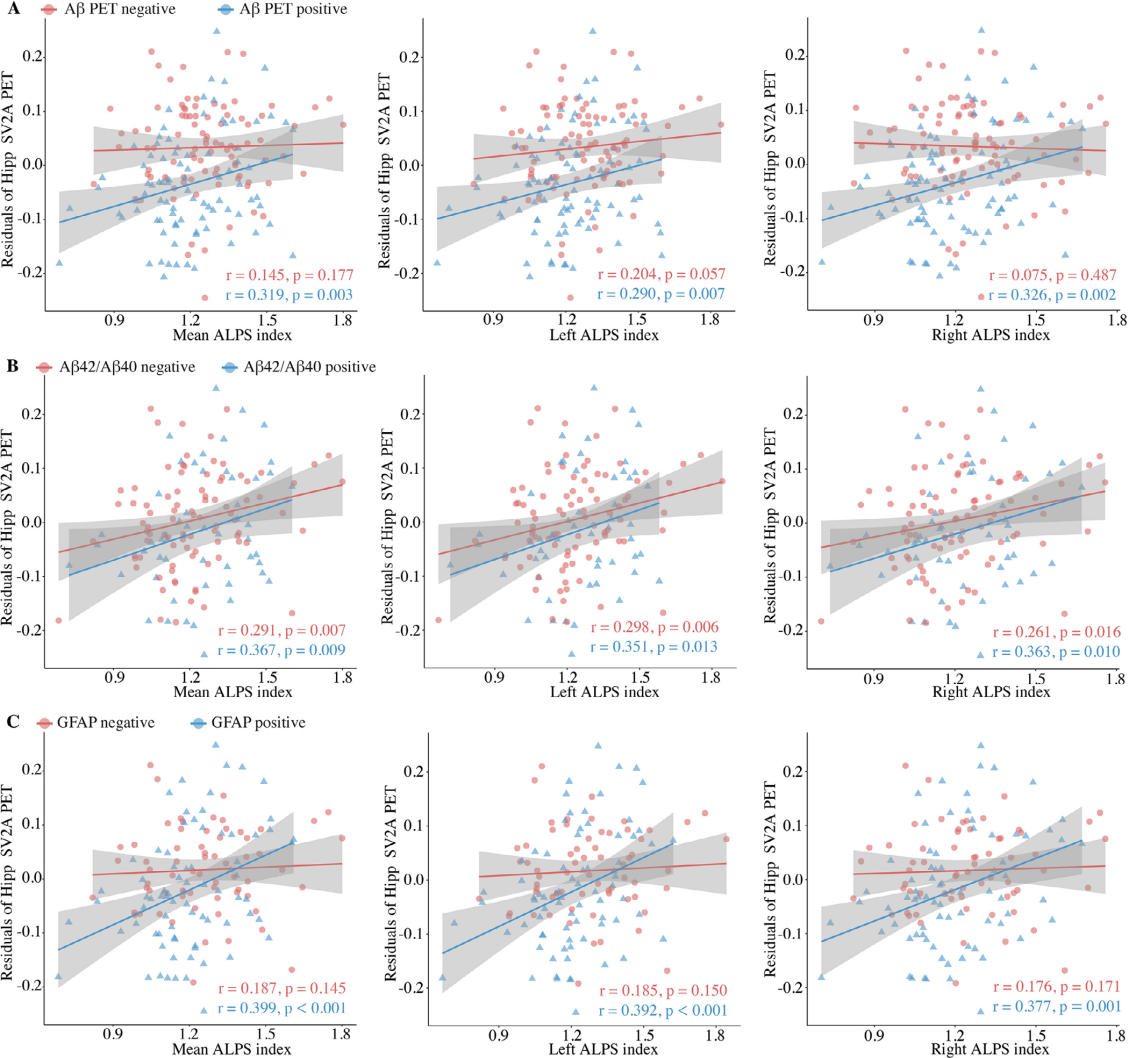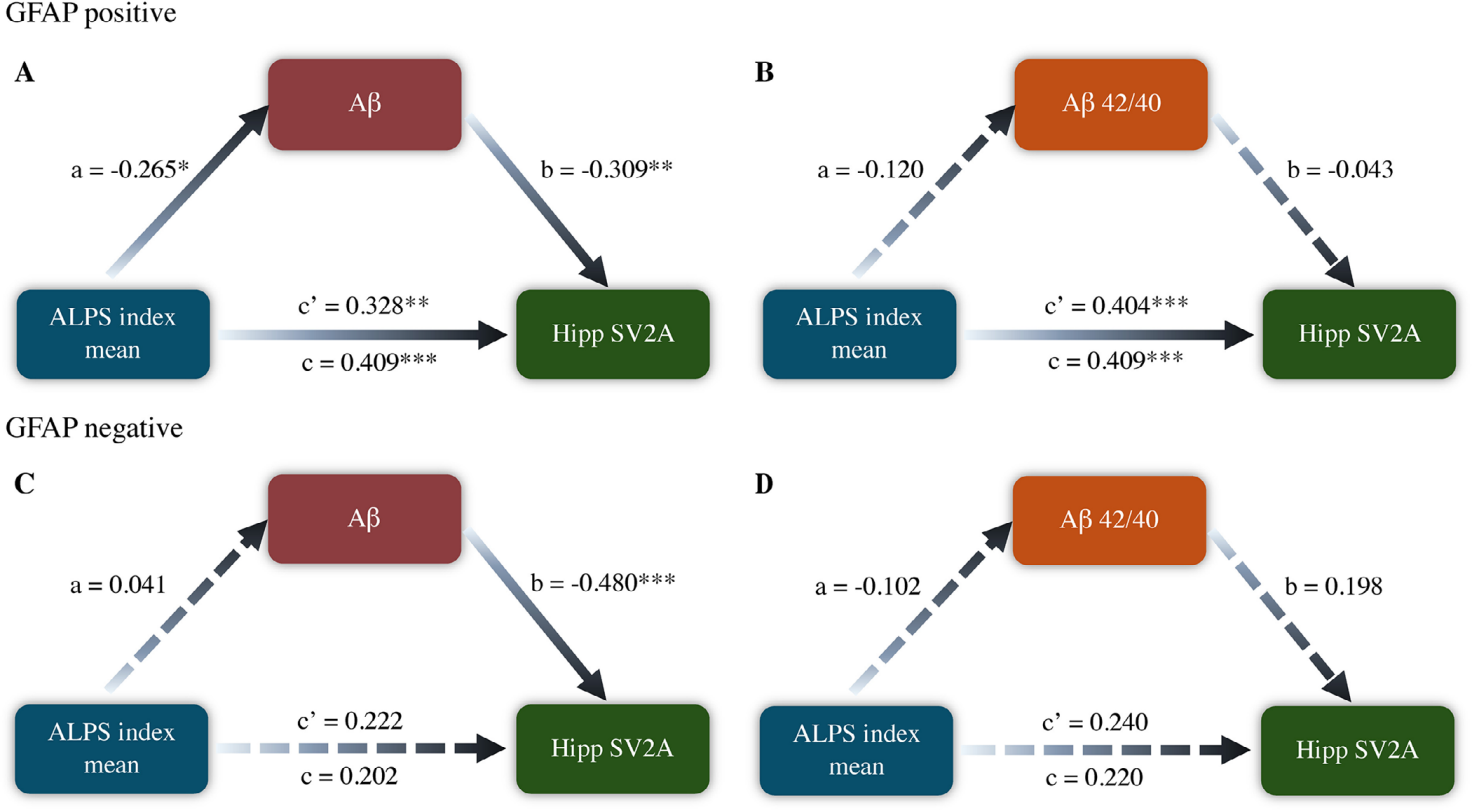# Impaired glymphatic function is associated with synaptic loss by SV2A PET in Alzheimer's Disease

**DOI:** 10.1002/alz70856_101757

**Published:** 2025-12-24

**Authors:** Kun He, Binyin Li, Qi Huang, Fang Xie

**Affiliations:** ^1^ Huashan hospital affiliated to fudan university, Shanghai, shanghai, China; ^2^ Ruijin Hospital affiliated to Shanghai Jiaotong University School of Medicine, Shanghai, Shanghai, China; ^3^ Department of Nuclear Medicine & PET Center, Huashan Hospital, Fudan University, Shanghai, Shanghai, China; ^4^ Huashan Hospital, Fudan University, Shanghai, Shanghai, China

## Abstract

**Background:**

Glymphatic system impairment is an important pathological change in Alzheimer's Disease (AD). However, there are few studies on the correlation between glymphatic system impairment and synaptic density reduction.

**Method:**

We utilized diffusion tensor imaging analysis along the perivascular space (DTI‐ALPS) index as a biomarker of the glymphatic system. Participants underwent synaptic density and amyloid‐β (Aβ) PET imaging, and peripheral plasma Aβ42, Aβ40, and GFAP were measured.

**Result:**

The ALPS index significantly correlates with SV2A PET after controlling for age, sex, education, and cognitive status. ALPS index had significantly positive associations with residuals of hippocampus SV2A PET SUVr in participants with positive cerebral Aβ deposition or higher plasma GFAP concentration. In the GFAP‐positive group, cerebral Aβ plaque significantly mediated the relationship between ALPS and hippocampal synaptic density.

**Conclusion:**

Glymphatic system impairment is significantly correlated with loss of synaptic density. This association can be influenced by GFAP and cerebral Aβ plaque.